# Melanins from the Lichens *Lobaria pulmonaria* and *Lobaria retigera* as Eco-Friendly Adsorbents of Synthetic Dyes

**DOI:** 10.3390/ijms232415605

**Published:** 2022-12-09

**Authors:** Anna Rassabina, Venera Khabibrakhmanova, Vasily Babaev, Amina Daminova, Farida Minibayeva

**Affiliations:** 1 Kazan Institute of Biochemistry and Biophysics, FRC Kazan Scientific Center of RAS, 2/31, Lobachevsky Str., Kazan 420111, Russia; 2 Arbuzov Institute of Organic and Physical Chemistry, FRC Kazan Scientific Center of RAS, 8, Academician Arbuzov Str., Kazan 420088, Russia

**Keywords:** lichens, melanins, FT-IR, synthetic dyes, adsorbents

## Abstract

Synthetic dyes are widely used in the industry; they are chemically stable, difficult to neutralize, and therefore they are a threat to the environment when released into wastewaters. The dyes have a significant impact on plant performance by impairing photosynthesis, inhibiting growth, and entering the food chain and may finally result in the toxicity, mutagenicity and carcinogenicity of food products. Implementation of the dark piment melanin for the adsorption of the synthetic dyes is a new ecologically friendly approach for bioremediation. The aim of the present work was to study the physico-chemical characteristics of melanins from the lichens *Lobaria pulmonaria* and *Lobaria retigera*, analyze their adsorption/desorption capacities towards synthetic dyes, and assess the capacity of melanins to mitigate toxicity of the dyes for a common soil bacterium *Bacillus subtilis*. Unique chelating properties of melanins determine the perspectives of the use of these high molecular weight polymers for detoxification of xenobiotics.

## 1. Introduction

Synthetic organic dyes are the largest group of all coloring substances. More than 100,000 commercially available synthetic organic dyes are widely used in the textile, tanning, printing, paper and plastic industries, the pharmaceutical and cosmetics industries as well as in food processing [1]. These dyes pose a threat to the environment when released into wastewater. In particular, they can cause a significant negative impact on plant performance by impairing photosynthesis, inhibiting growth, and entering the food chain and may finally result in the toxicity, mutagenicity and carcinogenicity of food products. Synthetic dyes are chemically stable; therefore, they are difficult to neutralize. In this regard, the use of various adsorbents to remove these dyes is an effective approach of remediation of wastewaters. The advantage of using biosorbents is due to their high efficiency, ease of technical implementation, and the possibility of multiple use of sorbents in the adsorption/desorption cycles. To date, biosorbents obtained from natural sources are of a particular interest. Unlike synthetic adsorbents, biosorbents practically do not cause detrimental effects on the environment [2,3].

At present, melanins, the dark pigments synthesized in animals, plants, fungi, and bacteria, are considered as perspective biosorbents. Melanins are high molecular weight hydrophobic polymers with intricate structural organization and chemical composition [4]. Pigments can be synthesized via the acetate-malonate and shikimate pathways in the reactions of oxidation and polymerization of phenolic and/or indolic compounds. As a result, different types of melanins such as eumelanin, allomelanin, pheomelanin, and others can be produced [5]. Melanins manifest high biological activities, including antioxidant, photo- and radioprotective activities, while they do not exhibit pronounced toxicity [6,7]. Moreover, due to their high reactivity, melanins demonstrate pronounced adsorption capacity towards heavy metal ions and toxic elements. For example, melanin extracted from micromycete *Amorphotheca resinae* can adsorb Pb, Cu, Cd, and Zn [8]. Melanin extracted from marine bacterium *Pseudomonas stutzeri* and functionalized by Fe/Cu impregnation can effectively bind As [9]. These micro-organisms with high content of melanin belong to extremophilic organisms, which are able to degrade oil products and use them for their own growth.

Lichens represent another example of organisms with high stress tolerance. They dominate over large areas of terrestrial ecosystems, for example as components of deserts, boreal, Arctic and Antarctic regions, therefore lichens can tolerate severe abiotic stresses. Lichens are symbiotic photosynthesizing organisms whose thalli are formed by mycobiont (fungi) and photobiont (algae and/or cyanobacteria) [10]. It seems likely that symbiotic co-existence of myco- and photobionts results in the synthesis of numerous secondary metabolites, which contribute to the high stress tolerance of lichens. Physico-chemical properties and biological activity of the unique lichen substances, terpenes, sterols, and fatty acids are well-studied [11,12,13,14]. However, only scarce information is available about physico-chemical characteristics of lichen melanins, although the involvement of these pigments in the tolerance of lichens to light stress has been documented [15]. To date, it is still unknown whether chemical composition, structural organization, and biological activity of lichen melanins differ from those of the melanins from other living organisms.

Earlier it was demonstrated that UV exposure of the green foliose lichen *Lobaria pulmonaria* can induce melanization of the upper cortex of thalli [15]. Lichen *Lobaria retigera* although being naturally dark-brown can also synthesize melanin in response to UV radiation [16]. The aim of the present work was to study how physico-chemical characteristics determine the capacity of the adsorption/desorption of synthetic dyes by melanins extracted from the lichens *L. pulmonaria* and *L. retigera*. Adsorption/desorption capacity of lichen melanins was studied using the water-soluble synthetic dyes that differ in their structure, charge, and size of molecule: thiazine dye Methylene Blue (MB), anthraquinone dye Remazol Brilliant Blue R (RBBR), indigoid dye Indigo Carmine (IC). To mathematically describe the adsorption equilibrium established during the interactions of dye molecules with melanins, the nonlinear models of Langmuir and Freundlich were applied. Based on the data on the desorption of the dyes bound to melanins, the optimal parameters for their regeneration were established. To compare adsorption/desorption capacities of melanins extracted from two lichens, the physico-chemical characteristics, such as content of the elements and main functional groups, as well as the structural organization were analyzed. Furthermore, the perspectives of application of melanins for remediation of wastewaters was assessed based on their capacity to tightly bind adsorbed dyes and as a consequence to reduce their biological toxicity towards a common soil bacterium *Bacillus subtilis*.

## 2. Results and Discussion

### 2.1. Physico-Chemical Characteristics of Melanins from Lichens L. pulmonaria and L. retigera

#### 2.1.1. Melanin Yield and Qualitative Analysis

The melanins extracted from two lichens *L. pulmonaria* and *L. retigera* yielded in average ca. 3% of the dry weight. Compared to other natural sources of melanins, the amount of extracted pigments from lichens is higher than that from micro-organisms but less compared to fungi [17].

Analysis of qualitative reactions of melanins demonstrated that in the presence of H_2_O_2_ melanins became discolored and in the presence of KMnO_4_ the color changed from dark brown to green. The addition of 10% FeCl_3_ to melanin containing solution led to precipitation of a sediment, which was further dissolved in the presence of excess FeCl_3_. The results of these qualitative reactions indicate the presence of phenolic fragments, typical for melanins [18].

#### 2.1.2. Elemental Analysis

Analysis of the elemental composition showed that lichen melanins differ in the content of C, H, N and O from the standard, a commercial eumelanin from *Sepia officinalis* (Table 1). Melanins from *L. pulmonaria* and *L. retigera* contained similar content of C and H, 48% and 6% H on average, respectively. However, the content of N significantly differed in two melanins. In melanin from *L. retigera* the content of N was more than doubled compared to that in melanin from *L. pulmonaria*. It can be suggested that this difference in the content of N is due to the differences in the photobionts of two lichen species. *L. pulmonaria* has two photobionts—non-N-fixing alga *Symbiochloris* and N-fixing cyanobacterium *Nostoc*, while *L. retigera* has only cyanobacterial photobiont *Nostoc* with high N-fixing activity [19].

Ratio of C/O in the structure of lichen melanins was approximately 1.3 times higher than that in the standard, indicating a higher content of oxygen-containing fragments. A high degree of aromaticity (C/H) of lichen melanins, especially melanin from *L. pulmonaria*, suggests that condensed systems occupy a large proportion in their structures (Table 1). However, for commercial eumelanin from *S. officinalis* the degree of aromaticity was much higher. The C/N ratio in melanin from the lichen *L. retigera* and from *S. officinalis* was similar, and therefore, this pigment can be attributed to eumelanin. Melanin from *L. pulmonaria* differed by a lower content of N, which is typical for allomelanins [20], suggesting a possible mixed chemical nature of this melanin.

#### 2.1.3. X-ray Fluorescence Analysis

X-ray fluorescence analysis revealed the presence of metals Fe, Al, Zn, Cu in lichen melanins (Supplementary Table S1). Melanin from *L. retigera* contained Fe, Al and Cu 1.5, 1.6 and 1.3 times more than those in melanin from *L. pulmonaria*. Pigment from *L. retigera* contained Zn 3.9 times less than in melanin from *L. pulmonaria*. It is known that in fungal melanins Fe and Al are bound by hydroxyl, amine, imine, and acetate groups, while Zn and Cu are bound by carboxyl groups [21]. It should be noted that Cu is an important cofactor for enzymes involved in the synthesis of melanins, including tyrosinases and laccases [22].

#### 2.1.4. FT-IR Spectroscopy

The complex chemical composition of melanins extracted from lichens was further confirmed by FT-IR spectroscopy.

**Figure 1:**
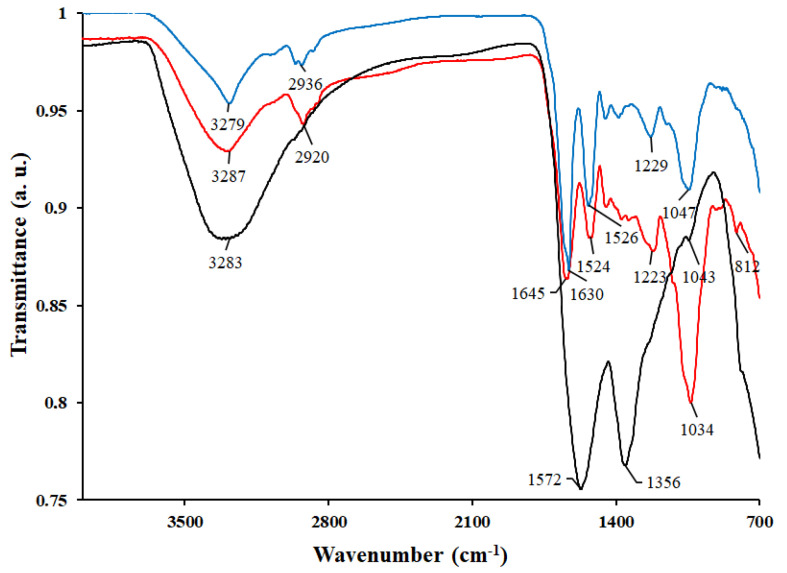
FT-IR spectra of melanins extracted from *L. pulmonaria* (red line); *L. retigera* (blue line); *S. officinalis* (black line).

The broad absorption band at 3279–3283 cm^−1^ was attributed to the stretching vibrations of the –OH и-NH groups in structure of melanins (Figure 1). The absorption peak at 2852–2853 cm^−1^ could be assigned to stretching vibration of aliphatic group. Absorption bands of the carboxyl groups at 1730 cm^−1^ and 1680 cm^−1^ were not observed, probably because of their low content and/or overlapping with an intensive neighboring absorption band in the region 1645–1630 cm^−1^, which was attributed to the vibrations of aromatic ring C=C. The band at 1500–1400 cm^−1^ was assigned to the bending vibrations of –N–H and stretching vibration of –C–N indole fragment. The peak at 1250–1120 cm^−1^ was related to the C–O group. The intensive peak centered at 1034 cm^−1^ (for melanin from *L. pulmonaria*) or 1047 cm^−1^ (for melanin from *L. retigera*) (Figure 1) could be an indication of the C–H in-plane of aliphatic structure characteristic of melanin pigment, aromatic CH groups, alkene CH substitution/conjugated systems, and/or symmetric contraction vibration of C–O–C bond [23,24,25]. The presence of a simple ether bond (C–O–C) and peak at approximately 890 cm^−1^ and 900 cm^−1^ in the melanin from *L. pulmonaria* indicates the occurrence of polysaccharides with *ß*-type bond [26]. In the FT-IR spectra of melanins from lichens, the absorption bands were observed in areas characteristic of melanins from various natural sources [4,27,28,29]. However, in comparison with melanin from *S. officinalis*, lichen melanins lacked a pronounced absorption peak at 1356 cm^−1^ because of the presence of C=O in aromatic conjugate structures, as well as an absorption peak at *c.* 1040 cm^−1^ (Figure 1). Therefore, these results suggest that among functional groups discovered in lichen melanins, hydroxyl (–OH) and carboxyl (–COOH) groups are the main functional groups, which can be involved in the adsorption of dyes.

#### 2.1.5. Chemical Analysis of Melanins

Further differences between two melanins were shown in the content of polysaccharides (Table 2). It is known that melanin binds strongly to the polysaccharides of the cell tissue of the fungus [30]. It can be suggested that polysaccharides, which are structurally linked to melanins, can increase their sorption activity towards metals and dyes.

These data are consistent with the results of quantitative determination of these functional groups by the titration in alkali solutions of melanins (Table 2). Indeed, the content of –OH groups was 1.8 times higher in melanin from *L. pulmonaria*, whereas the content of –COOH groups was 2.7 times higher in melanin from *L. retigera*. Interestingly, the content of –OH groups in the eumelanin from *S. officinalis* was similar to that in melanin from *L. pulmonaria*, while the content of –COOH groups in the eumelanin from *S. officinalis* was similar to that in melanin from *L. retigera*, indicating the differences in reactivity of melanins including their chelating abilities.

#### 2.1.6. Zeta (ζ) Potential of Melanins

Using dynamic light scattering (DLS) it was demonstrated that melanins possess negative zeta (ζ) potential. In melanin from *L. pulmonaria* the zeta (ζ) potential was −32.73 ± 9.71 mV, while in melanin from *L. retigera* it was −12.63 ± 7.39 mV. For melanin from *L. pulmonaria* (ζ) potential was 2.6 times higher compared to that in melanin from *L. retigera*, which correlates with a large number of functional groups in its structure (Table 2). The (ζ) potential corresponds to the surface potential and therefore it may influence the processes of adsorption/desorption of dye ions.

Taken together, data suggest that melanins from two lichen species demonstrate differences in the degree of aromaticity, the ratio of C/N, metal chelating abilities, and the number of –OH and –COOH groups. These differences might determine the discrepancies in their physico-chemical properties, including the ability of adsorption/desorption of synthetic dyes.

### 2.2. Adsorption Studies

#### 2.2.1. Chemical Structure of Applied Synthetic Dyes

**Figure 2:**
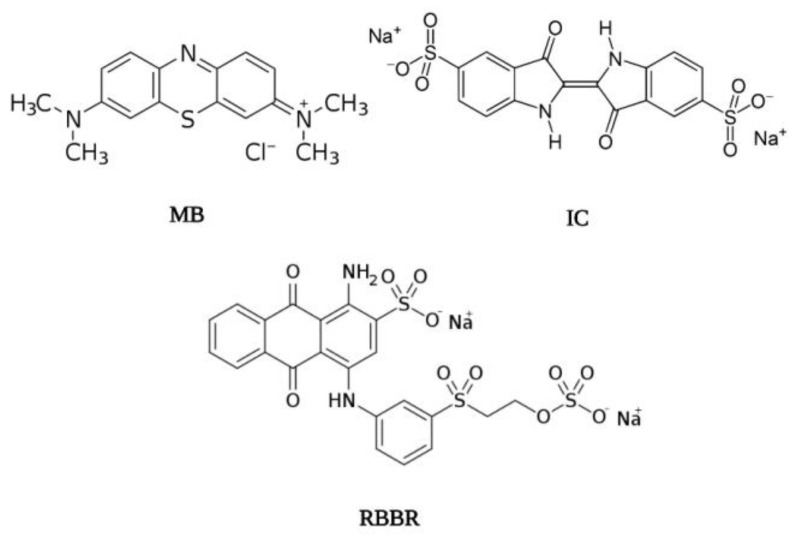
Chemical structure of synthetic dyes MB, RBBR and IC.

To assess the adsorption capacity of melanins from lichens *L. pulmonaria* and *L. retigera* water-soluble synthetic dyes MB, RBBR and IC, differing in the charge and molecular weight were chosen. The structure of synthetic dyes is shown in Figure 2. It is known that thiazine dye MB is widely used for dyeing cotton, silk, and wood [31]. This is a cationic dye and therefore it can participate in electrostatic interactions, and at the same time, it is capable of hydrophobic interactions. RBBR dye belongs to active anthraquinone dyes and is widely used in the textile and cotton industries [32]. It is an anionic dye containing SO_3_¯ groups. The poly-conjugate structure of the dye ensures its affinity with non-polar compounds [33]. Indigoid dye IC is used in the textile and food industries [34]. The presence of two SO_3_¯ groups provides anionic properties to the dye, and the presence of substituted heterocyclic fragments in the main structure ensures its participation in the intermolecular interactions [35,36,37].

To reduce the toxicity of synthetic dyes in ecosystems [38,39], it is crucial to develop an effective protocol of remediation of wastewaters, including adsorption of dyes. For this purpose, adsorption of dyes by melanins from lichens represents a promising approach.

#### 2.2.2. Evaluation of Adsorption Capacity of Lichen Melanins

The study of the adsorption capacity of lichen melanins towards synthetic dyes included the study of adsorption under static conditions using model solutions of dyes at a constant temperature. In the experiment, the concentration of dyes varied, while a concentration of melanin in the solution remained constant. The adsorption capacity of melanins from two lichens in relation to synthetic dyes was markedly different (Table 3).

At the maximum dye content in the solution (300 μg), adsorption of MB, RBBR, and IC by melanin from *L. pulmonaria* was 2, 1.7, and 3.2 times greater than that by melanin from *L. retigera*. Probably, the higher adsorption capacity of melanin from *L. pulmonaria* was due to the high content of –OH functional groups and polysaccharides (Table 2).

**Figure 3:**
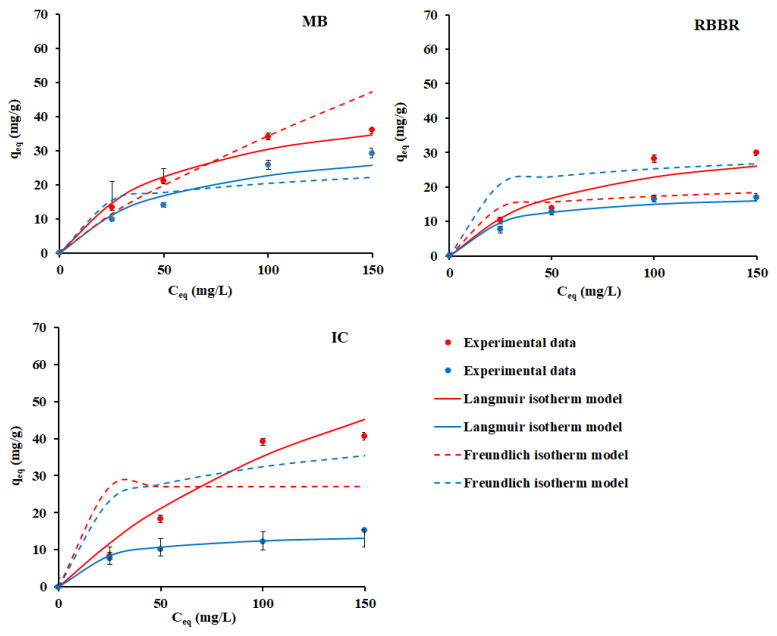
Langmuir and Freundlich isotherm models of adsorption of synthetic dyes MB, RBBR and IC by melanins from *L. pulmonaria* (red line); *L. retigera* (blue line) at 298.1 °K. Solid line corresponds to the Langmuir isotherm model, dotted line corresponds to the Freundlich isotherm model.

Langmuir and Freundlich isotherm models were used to describe the mechanism of adsorption of synthetic dyes by lichen melanins. These models are widely adopted for investigating the relationship between adsorption capacity of adsorbent and equilibrium adsorbent concentration in an aqueous solution [40]. Adsorption isotherms are presented in Figure 3.

The main parameters, including the constants of the Langmuir and Freundlich equations, were calculated and presented in Supplementary Table S2. Values q_eq_ using the Langmuir and Freundlich models (Section 3.4.2) were consistent with the experimental data (Figure 3). According to the Langmuir model, the formation of a monomolecular layer on the surface of the sorbent does not involve interactions between the adsorbate particles [41,42]. According to the Freundlich model, adsorption occurs on an energetically heterogeneous surface [43]. According to the type of isotherm and the obtained values (R^2^), the adsorption of synthetic dyes MB and RBBR by lichen melanins could be correctly described by the Freundlich isotherm model (Figure 3; Supplementary Table S2), due to the presence of the heterogeneous adsorption centers in the melanin structure.

The maximum dye adsorption capacity (q_max_) was calculated according to the Langmuir model. For melanin from *L. pulmonaria* it was 47.63, 36.03, 92.96 mg/g, while for melanin from *L. retigera* it was 34.97, 18.28, 14.72 mg/g for MB, RBBR and IC, respectively (Supplementary Table S2). These data suggest that the melanin from *L. pulmonaria* demonstrates higher dye adsorption capacity. Presumably, MB adsorption is caused by electrostatic interactions between the -NH group dyes and negatively charged functional groups of melanin. Total amount of negatively charged functional groups in the melanin of *L. pulmonaria* was 5% more than that in the melanin of *L. retigera* (Table 3). Adsorption of anionic dyes is associated with the presence of hydrophilic centers in the structure of melanin [44]. RBBR contains both cationic and anionic functional centers, which can interact with the aromatic skeleton of melanins via *π*–*π* or n–*π* interactions, and therefore the mechanisms of adsorption of this dye by lichen melanins are more complex. A significant limitation of the effective RBBR adsorption is high molecular weight and a complex branched structure of this dye [45]. The highest adsorption capacity melanin from *L. pulmonaria* demonstrates towards the anionic dye IC (92.96 vs. 14.72 mg/g for the melanin from *L. retigera*) that can be explained by interactions of the dye with the aromatic skeleton of melanins and a small size of its molecule. Moreover, the presence of polysaccharides in the structure of melanins (Figure 1; Table 2) can facilitate the adsorption of dyes.

Therefore, the melanins from the lichens *L. pulmonaria* and *L. retigera* can adsorb synthetic dyes. The efficiency of adsorption is determined by the chemical characteristics of a dye and physico-chemical properties of lichen melanins.

#### 2.2.3. Regeneration Studies

In biotechnological practice, an important property of adsorbents is the possibility for their reuse in adsorption and desorption cycles [46]. The desorption of synthetic dyes was studied under constant stirring and successive treatments with water, heating at 30 °C, and pH change (Table 4).

It was found that the binding strength of dyes by melanins varies depending on the chemical structure of a dye and exposure conditions. The RBBR, which is characterized by a complex branched structure and a low total charge, when treated with water, was actively released into the solution (9–13% on average) from both melanins. When treated with heat and pH 3, desorption of RBBR is negligible—no more than 1% from *L. pulmonaria* melanin, no more than 3% from *L. retigera*. These results indicate that RBBR is strongly bound by melanins and cannot be easily desorbed.

Differences in the structural organization and reactivity of melanins depending on the lichens from which they were extracted determined their different retention capacity with respect to the anionic dye IC and the cationic dye MB. Following each treatment melanin from *L. retigera* released more dye compared to melanin from *L. pulmonaria* (Table 4). This may be due to the high content of negatively charged carboxyl groups in melanin from *L. retigera* (2.6 times) (Table 2) and the lower content of polysaccharides. Melanin from *L. retigera* demonstrated a 4.3 times higher desorption capacity for MB during incubation in water compared to that for melanin from *L. pulmonaria* (Table 4). Gentle heating did not facilitate release of the dye, while acidification of the incubation medium greatly (10 times) increased the release of MB. This may be caused by the transfer to the main status (non-dissociated) of negatively charged carboxyl groups of melanin from *L. retigera* and their disability to interact with dye molecules.

Altogether, these data suggest that melanin from *L. retigera* has a higher capacity for desorption of all synthetic dyes applied in this study. It is important to note that in the alkali solution (pH 10) melanins were fully dissolved and dyes released (Table 4). This provides a possible mechanism for regeneration of melanins including the alkali dissolvement of melanin/dye complex followed by membrane filtration, removal of dyes and recovery of purified melanins for further reuse.

#### 2.2.4. Reducing the Biological Toxicity of Synthetic Dyes by Their Adsorption

Synthetic dyes, when released into natural ecosystems, have a pronounced toxic effect on living organisms [47,48,49,50]. We analyzed how adsorption of dyes by melanins can mitigate their biological toxicity using nonpathogenic soil bacteria *B. subtilis*. These micro-organisms occupy an important niche in the microbial consortium of natural systems, and the impact of dyes on their viability can shift the ecological balance [50,51]. In our preliminary experiments, we tested the effects of lichen melanins on the growth of bacterium *B. subtilis*. It was found that melanins on their own do not have a significant inhibitory or stimulating effect on bacterial growth. Here, it was shown that synthetic dyes MB and RBBR cause an inhibitory effect on the growth of *B. subtilis* (Table 5; Supplementary Figure S1). In contrast to MB and RBBR, IC did not show a pronounced toxic effect on bacteria (data are not present). In the presence of melanins extracted from lichens *L. pulmonaria* and *L. retigera* the negative effect of synthetic dyes on bacterial flora was less (Supplementary Figure S1). For example, the inhibitory effect of MB on bacterial growth was reduced by ca. 30% when melanins containing the same amount of adsorbed dye were applied. When evaluating the toxic effect of RBBR on *B. subtilis*, it was found that RBBR adsorbed by melanin from *L. pulmonaria* does not display a bactericidal effect, while RBBR adsorbed by melanin from *L. retigera* showed a weak inhibitory effect on the growth of *B. subtilis*.

The results obtained in biological studies using *B. subtilis* demonstrated that effectiveness of lichen melanins to reduce toxicity of synthetic dyes is determined by their physico-chemical characteristics and adsorption properties. Indeed, melanin from *L. pulmonaria* with higher adsorption capacity (Table 3) and lower desorption capacity (Table 4) could more effectively reduce the toxicity of synthetic dyes (Table 5). Melanin *L. retigera* was not able to effectively retain dyes and reduce the toxicity of synthetic dyes; however, it showed higher desorption capacity (Table 4).

## 3. Materials and Methods

### 3.1. Chemicals/Reagents

Dyes MB, RBBR, IC; melanin from *S. officinalis* were analytical grade (Sigma-Aldrich, St. Louis, MO, USA). H_2_O_2_, KMnO_4_, FeCl_3_, NaOH, LiOH, BaCl_2_, (CH3COO)_2_Ca, methyl red, phenolphthalein and organic solvents were of reagent grade (Russia).

### 3.2. Extraction and Purification of Melanins from Lichens L. pulmonaria and L. retigera

Melanins were extracted from lichens *L. pulmonaria* and *L. retigera*. Lichen *L. pulmonaria* was collected from the bark of oak trees at Langangen, South Norway (59.096° N; 9.792° E) and *L. retigera* from the bush branches in Fort Nottingham in the province KwaZulu-Natal, South Africa (29.414° S; 29.913° E). Dry lichen thalli were crushed to a powder with the addition of liquid nitrogen, then powder (5 g) was incubated with 50 mL 2 M NaOH for 24 h, and extract centrifuged at 150 rpm (Hermle, Gosheim, Germany) for 10 min. The supernatant was acidified by adding 2 M HCl, incubated for 12 h at room temperature, and centrifuged at 150 rpm for 10 min. The precipitate containing melanin was rinsed with distilled water, then successively purified with chloroform, ethyl acetate and acetone (2 × 10 mL). The purified melanin was lyophilized. Yield of melanin from *L. pulmonaria* was 40 ± 5 mg/g DW and melanin from *L. retigera*—42 ± 8 mg/g DW. Melanins extracted from lichens were brown and dark brown powders. Melanin powders were sifted and fractions no larger than 1 mm were stored at −20 °C until use for analyses.

### 3.3. Physico-Chemical Characteristics of Melanins

#### 3.3.1. Elemental Composition and Content of Polysaccharides

Solutions of melanins (0.4 mg/mL) were prepared in distilled water with the addition of 10% NH_4_OH to pH 7.0. Identification of melanins was carried out using qualitative reactions with H_2_O_2_, KMnO_4_ and FeCl_3_ [4,51]. Elemental composition of melanins (C, N, H) determined using an analyzer EuroEA 3028-HT-OM (Eurovector SpA, Pavia, Italy) [52], the oxygen content was determined by calculation.

Content of ash including metals was assessed by X-ray fluorescence analysis (XRF) using an energy-dispersive fluorescent X-ray spectrometer EDX-800HS2 (Shimadzu, Kyoto, Japan). The measurements were carried out under the following conditions: maximum input voltage 50 kV, maximum current 1 mA, helium atmosphere. XRF analysis of melanins was processed using EDX software.

The total polysaccharides content in the extracted melanins was estimated by the anthrone method [53] using glucose as a standard.

#### 3.3.2. Functional Groups

The functional groups of melanins were analyzed using Fourier-transform infrared (FT-IR) spectroscopy (IR-Affinty1, Shimadzu, Kyoto, Japan). The measurements were carried out using following parameters: wavenumber range 4000–700 cm^–1^, the spectral resolution fixed to 4 cm^−1^, the number of scans to 32. The FT-IR spectra of melanins were processed using OriginPro 8 software.

Quantitative analysis of –OH and –COOH groups in melanins was carried out by LiOH titration according to the protocol with modification [54]. The number of –OH groups was calculated using the equation:

(1) $$\left[\mathrm{OH}\right]=\frac{\left(a^{0}-a\right)\times f\times 1.7\times 1.25}{A}\times 100\%$$

where a, a_0_—volumes 0.1 M LiOH, used for titration in the working and blank experiments, respectively (mL); f—factor 0.1 M LiOH; 1.7—weight of –OH groups equivalent to 1 mL 0.1 M LiOH (mg); 1.25—full volume conversion factor; A—weight of melanin sample (mg). The number of –COOH groups was calculated using the equation:

(2) $$\left[\mathrm{COOH}\right]=\frac{\left(a-a^{0}\right)\times f\times 1.25\times 0.85}{A}\times 100\%$$

where a, a_0_—volumes 0.05 M LiOH, used for titration in the working and blank experiments, respectively (mL); f—factor 0.05 M LiOH; 0.85—weight of –COOH groups, equivalent to 1 mL 0.05 M LiOH (mg); 1.25—full volume conversion factor; A—weight of melanin sample (mg).

#### 3.3.3. Colloidal Properties

The zeta (ζ) potential determination of samples was carried out by a Malvern ZS Nanosizer (Malvern Instruments Ltd., Malvern, UK) Dynamic Light Scattering (DLS). Sample preparation, included the preparation of melanin solutions at a concentration of 0.4 mg/mL, filtration through a membrane filter (45/13 Chromafill Xtra, 0.45 µm, Düren, Germany).

### 3.4. Adsorption Properties of Melanins

#### 3.4.1. Adsorption of Synthetic Dyes

Melanin powder (500 µg) was incubated with 3 mL of dyes in concentrations of 25, 50, 100 mg/L at 25 °C with continuous stirring at 100 rpm for 30 min (BioSan, Riga, Latvia). Solutions were then centrifuged at 240 rpm for 10 min (Eppendorf, Hamburg, Germany). The absorbance of the supernatant was measured at dye absorption maxima (663 nm for MB; 595 nm for RBBR; 612 nm for IC) using a UV-1900 spectrophotometer (Shimadzu, Kyoto, Japan). Calculation of the amount of dye adsorbed by melanin was determined using the experimentally obtained calibration curves of the dependence of the optical density of dye solutions on their concentration (Supplementary Table S3). The amount of dye adsorbed was calculated using the following equation:

(3) $$q=\left(C_{0}-C_{1}\right)\frac{V}{W}$$

where q—amount of dye adsorbed on melanin (mg/g); C_0_—the initial concentration of the dye in the solution (mg/L); C_1_—dye concentration in solution after adsorption (mg/L); V—volume of the solution (L); W—the weight of melanin taken for analysis (g).

#### 3.4.2. Construction of Adsorption Isotherms

To determine the maximum of adsorption capacity of melanins, adsorption isotherm according to the models of Langmuir and Freundlich were constructed. Using experimental data (Section 3.4.1), calculations were carried out using the formulas:

(4) $$q_{e}=q_{\max}K_{L}C_{e}/\left(1+K_{L}C_{e}\right)$$

(5) $$q_{e}=K_{F}C_{e}^{1/n}$$

where q_max_—maximum adsorption capacity (mg/g); K_L_—Langmuir constant; C_e_—initial amount of dye (mg/L); K_F_—Freundlich constant; 1/n—the value of the adsorption index.

### 3.5. Desorption of Synthetic Dyes Adsorbed by Melanins

Based on the experiments described in Section 3.4.1, melanins (500 µg) after incubation with 3 mL dye (50 mg/L) were sequentially washed according to the following scheme: (1) incubation in distilled water at 100 rpm for 30 min at room temperature and centrifugation at 240 rpm for 10 min three times, (2) resulting pellets were incubated in distilled water at 100 rpm and a temperature of +30 °C for 30 min and centrifuged, (3) resulting pellets were incubated in water solutions with pH 3 and pH 11 at 100 rpm for 30 min and centrifuged. Optical density of the resulting supernatants was measured as described in Section 3.4.1. The calculation of the desorption of the dye adsorbed with melanin was performed according to the equation:

(6) $$D\;=\frac{q_{a}}{q_{d}}\times 100\%$$

where q_a_—amount of dye in solution (μg); q_d_—initial amount of dye in solution (μg).

### 3.6. Biotesting of the Toxicity of Synthetic Dyes Adsorbed by Melanins

Toxicity of synthetic dyes absorbed by melanins was tested using a common soil bacterium *Bacillus subtilis* by the disk-diffusion method. The bacterial inoculum was grown in Luria-Bertani medium (LB) [55] in a shaker-incubator ES-20/60 (BioSan, Riga, Latvia) at 170 rpm, 37 °C for 16 h, and after that 100 µL of bacterial culture was plated on 1.5% agar gel in Petri dishes. The dye solution was applied to sterile paper discs (10–50 µg/disk) and incubated at 37 °C for 12 h. Melanin (150 µg) containing 50 µg of adsorbed dye was dispersed in the calculated volume of distilled water; the resulting solution was applied to a disk. Retardation zone of bacterial growth was measured (in mm). Toxicity of each dye adsorbed by melanins was assessed using the same protocol.

### 3.7. Statistical Analysis

The experiments were carried out in three biological and at least three analytical replicates. Calculations were made in Microsoft Excel 2016. The results of the experiments were statistically processed and presented in the work at a confidence level *p* = 0.95, n = 3 (n is the number of experiments).

## 4. Conclusions

Various physico-chemical treatments, such as ion exchange, reverse osmosis, and advanced oxidation processes, are used to remove toxic compounds from wastewater. Adsorption is one of the most effective methods used in treatment [1]. The results obtained in this study demonstrate the effectiveness of lichen melanins in absorbing and reducing the toxicity of synthetic dyes. A comparative analysis of melanins extracted from the lichens *L. pulmonaria* and *L. retigera* demonstrated differences in their chemical composition, structure, and the content of functional groups. Melanin from the lichen *L. pulmonaria* has a high degree of aromaticity, low content of N, high content of Zn, which correlates with high content of hydroxyl groups. Melanin from the lichen *L. retigera* is characterized by high content of N and based on the C/N ratio melanin from the lichen *L. retigera* can be attributed to eumelanin. High content of Fe and Al correlates with a bigger amount of carboxyl groups. Given their physico-chemical properties, melanin from *L. pulmonaria* is more effective in terms of the adsorption capacity and retention ability of synthetic dyes compared to melanin from *L. retigera*. Melanin from the lichen *L. pulmonaria* showed great heterogeneous adsorption capacity for the cationic dye MB, anionic dye RBBR and monolayer adsorption capacity for IC. The adsorption properties of melanin can be enhanced due to the presence of polysaccharides in its composition. During the adsorption process, dyes easily interact with functional groups of melanins, as well as with their aromatic polyconjugated structures through hydrogen bonds and electrostatic and donor-acceptor interactions. As a result of dye sorption, melanins can reduce their toxicity to living organisms, e.g., nonpathogenic soil bacteria *B. subtilis*.

In the future, based on the fundamental knowledge of physico-chemical properties of lichen melanins, melanin-like materials can be synthesized to develop new ecologically friendly and highly efficient reusable remediation technologies. Application of lichen melanin is a basis for a new ecologically friendly approach for remediation.

## Figures and Tables

**Table 1:** The element content of C, H, N, O and atomic ratio in the melanins.

Melanins	C (%)	H (%)	N (%)	O (%)	Atomic Ratio		
					C/O	C/H	C/N
*L. pulmonaria*	49.62 ± 0.03	5.78 ± 0.10	4.44 ± 0.12	40.16	1.65 ± 0.12	0.72 ± 0.11	13.04 ± 0.01
*L. retigera*	47.38 ± 0.10	6.13 ± 0.11	9.47 ± 0.12	37.02	1.71 ± 0.14	0.64 ± 0.11	5.84 ± 0.11
*S. officinalis*	44.62 ± 0.20	3.39 ± 0.01	7.60 ± 0.05	44.39	1.34 ± 0.07	1.10 ± 0.14	6.85 ± 0.23

**Table 2:** The content of polysaccharides and –OH, –COOH functional groups in the melanins.

Melanins	Content of Polysaccharides (%)	–OH (%)	–COOH (%)
*L. pulmonaria*	19.93 ± 0.60	14.54 ± 1.85	1.06 ± 0.01
*L. retigera*	14.08 ± 0.19	8.08 ± 1.28	2.66 ± 0.75
*S. officinalis*	-	16.15 ± 0.90	2.19 ± 0.10

**Table 3:** The adsorption capacity of melanins from *L. pulmonaria* and *L. retigera*.

Melanins	Amount of Dye (μg) ^1^	Amount of Adsorbed Dye (μg)		
		MB	RBBR	IC
*L. pulmonaria*	75	67.49 ± 1.00	51.57 ± 0.04	41.29 ± 2.30
	150	140.50 ± 2.26	64.46 ± 4.41	93.54 ± 1.89
	300	260.40 ± 1.98	140.53 ± 0.42	197.19 ± 4.56
*L. retigera*	75	49.53 ± 0.34	38.40 ± 0.14	38.07 ± 1.10
	150	70.15 ± 4.69	64.46 ± 1.05	50.39 ± 4.65
	300	129.21 ± 3.50	82.96 ± 0.21	60.97 ± 1.97

^1^ Initial amount of dye in the reaction medium.

**Table 4:** Desorption of synthetic dyes adsorbed by melanins from *L. pulmonaria* and *L. retigera*.

Melanins	Treatment	Desorption (D) of the Adsorbed Dye (%) ^1^		
		MB	RBBR	IC
*L. pulmonaria*	H_2_O	1.50 ± 0.12	9.14 ± 0.11	2.16 ± 0.21
	H_2_O, 30 °C	0.16 ± 0.05	0.89 ± 0.06	0.60 ± 0.59
	H_2_O, pH 3	1.91 ± 0.23	1.08 ± 0.80	0.89 ± 0.32
	H_2_O, pH 11	n.d. ^2^	n.d.	n.d.
*L. retigera*	H_2_O	6.48 ± 0.75	12.68 ± 1.10	16.06 ± 0.73
	H_2_O, 30 °C	1.42 ± 0.21	3.00 ± 0.10	3.82 ± 0.11
	H_2_O, pH 3	61.83 ± 0.04	2.46 ± 0.05	3.89 ± 0.55
	H_2_O, pH 11	n.d.	n.d.	n.d.

^1^ On the amount of adsorbed dye. ^2^ Not determined.

**Table 5:** Effects of melanins on the bacterial growth retardation caused by MB and RBBR.

Dyes	Content of Dye (µg/disk)	Zone of Growth Retardation (mm)	
		MB	RBBR
Dye without melanins	50	28.8 ± 1.8	10.9 ± 1.6
Dye adsorbed by melanin from *L. pulmonaria*		20.0 ± 0.8	0
Dye adsorbed by melanin from *L. retigera*		21.0 ± 0.8	8.3 ± 0.5

## Data Availability

Not applicable.

## Supplementary Materials

The following supporting information can be downloaded at: https://www.mdpi.com/article/10.3390/ijms232415605/s1.
