# Supplementary material for: Melanins from the Lichens Lobaria pulmonaria and Lobaria retigera as Eco-Friendly Adsorbents of Synthetic Dyes
Source: Int J Mol Sci. 2022 Dec 9;23(24):15605. doi: 10.3390/ijms232415605 (PMC9779828; doi:10.3390/ijms232415605)
Supplement: Supplementary file 1 [file ijms-23-15605-s001.zip › Supplementary data Table S3.pdf]

Supplementary Table S3. Summary table of equations of the calibration graphs of the dependence of optical density of dye solutions on concentration.

Dye	Concentration range (μg/mL)	Equation	R^2
**MB**	1–5	y=0.1818x	0.9433
**RBBR**	2.5–12.5	y=0.0605x	0.9995
**IC**	5–25	y=0.0445x	0.9999
