# Supplementary material for: Melanins from the Lichens Lobaria pulmonaria and Lobaria retigera as Eco-Friendly Adsorbents of Synthetic Dyes
Source: Int J Mol Sci. 2022 Dec 9;23(24):15605. doi: 10.3390/ijms232415605 (PMC9779828; doi:10.3390/ijms232415605)
Supplement: Supplementary file 1 [file ijms-23-15605-s001.zip › Supplementary data Table S2.pdf]

Supplementary Table S2. Isotherm model constants and their respective correlation coefficients for the adsorption of synthetic dyes by melanin from *L. pulmonaria* and *L. retigera.*

Melanins	Dye	Langmuir			Freundlich		
		q_{max} (mg g^{-1})	K_L (L/mg)	R^2	K_F (mg g^{-1})	1/n	R^2
L. pulmonaria	MB	47.63	0.018	0.975	0.91	0.788	0.971
	RBBR	36.03	0.017	0.839	9.07	0.185	0.952
	IC	92.96	0.005	0.812	26,28	0.010	0.951
L. retigera	MB	34.97	0.019	0.928	8.11	0.202	0.979
	RBBR	18.28	0.048	0.986	3.26	0.679	0.917
	IC	14.72	0.053	0.976	4.18	0.836	0.987
