# Supplementary material for: Melanins from the Lichens Lobaria pulmonaria and Lobaria retigera as Eco-Friendly Adsorbents of Synthetic Dyes
Source: Int J Mol Sci. 2022 Dec 9;23(24):15605. doi: 10.3390/ijms232415605 (PMC9779828; doi:10.3390/ijms232415605)
Supplement: Supplementary file 1 [file ijms-23-15605-s001.zip › Supplementary data Figure S1.pdf]

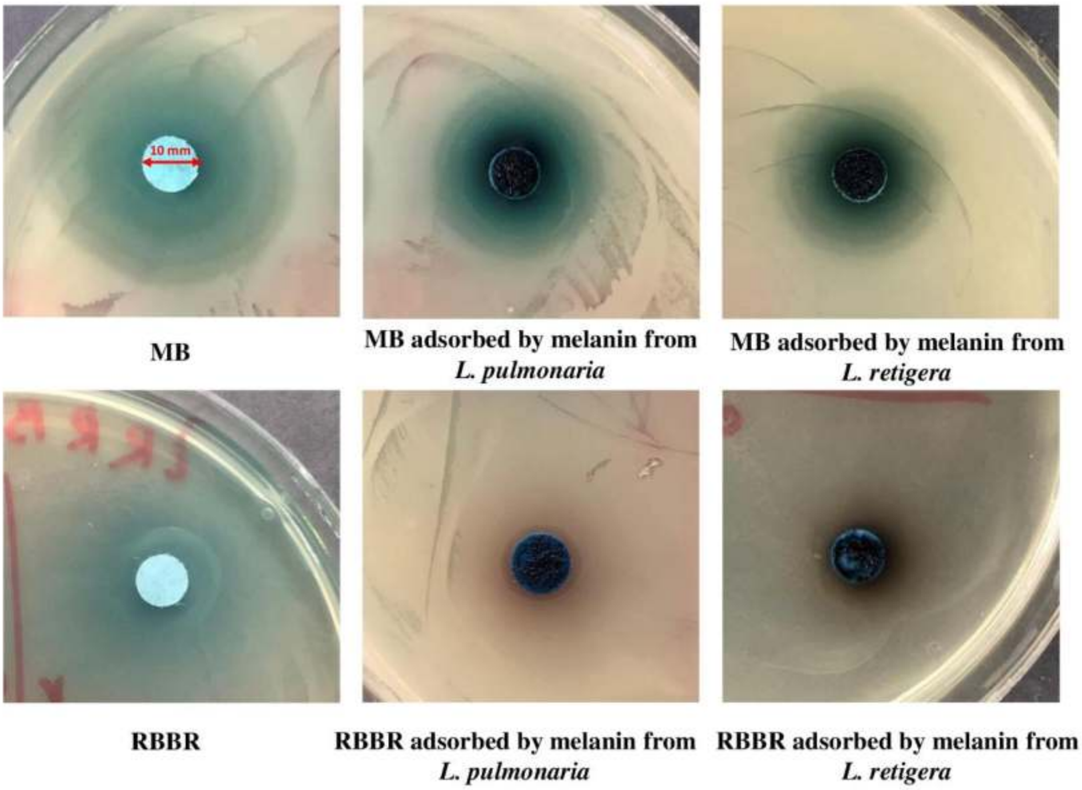

Supplementary Figure S1. Zones of growth retardation of soil bacterium *Bacillus subtilis* caused by 50 μg of synthetic dyes MB and RBBR and mitigation of growth retardation following the absorption of dyes by lichen melanins.
