# Supplementary material for: Melanins from the Lichens Lobaria pulmonaria and Lobaria retigera as Eco-Friendly Adsorbents of Synthetic Dyes
Source: Int J Mol Sci. 2022 Dec 9;23(24):15605. doi: 10.3390/ijms232415605 (PMC9779828; doi:10.3390/ijms232415605)
Supplement: Supplementary file 1 [file ijms-23-15605-s001.zip › Supplementary data Table S1.pdf]

Supplementary data for the manuscript by Rassabina et al. Melanins from the lichens *Lobaria pulmonaria* and *Lobaria retigera* as eco-friendly adsorbents of synthetic dyes

Supplementary Table S1. The percentage of metals in the ash of melanins.

Analyte	*L. pulmonaria*	*L. retigera*
**Fe**	8.24	12.68
**Al**	6.31	10.36
**Zn**	1.39	0.36
**Cu**	0.69	0.92
